# Combination of degradation pathways for naphthalene utilization in Rhodococcus sp. strain TFB

**DOI:** 10.1111/1751-7915.12096

**Published:** 2013-12-11

**Authors:** Laura Tomás-Gallardo, Helena Gómez-Álvarez, Eduardo Santero, Belén Floriano

**Affiliations:** Centro Andaluz de Biología del Desarrollo, CSIC-Universidad Pablo de Olavide-Junta de AndalucíaSeville, Spain

## Abstract

*Rhodococcus* sp. strain TFB is a metabolic versatile bacterium able to grow on naphthalene as the only carbon and energy source. Applying proteomic, genetic and biochemical approaches, we propose in this paper that, at least, three coordinated but independently regulated set of genes are combined to degrade naphthalene in TFB. First, proteins involved in tetralin degradation are also induced by naphthalene and may carry out its conversion to salicylaldehyde. This is the only part of the naphthalene degradation pathway showing glucose catabolite repression. Second, a salicylaldehyde dehydrogenase activity that converts salicylaldehyde to salicylate is detected in naphthalene-grown cells but not in tetralin-or salicylate-grown cells. Finally, we describe the chromosomally located *nag* genes, encoding the gentisate pathway for salicylate conversion into fumarate and pyruvate, which are only induced by salicylate and not by naphthalene. This work shows how biodegradation pathways in *Rhodococcus* sp. strain TFB could be assembled using elements from different pathways mainly because of the laxity of the regulatory systems and the broad specificity of the catabolic enzymes.

## Introduction

*Rhodococcus* sp. strain TFB is a Gram-positive bacterium able to metabolize a wide variety of aromatic compounds, such as phthalate (Tomás-Gallardo *et al*., 2006), tetralin (Tomás-Gallardo *et al*., 2009), naphthalene, benzene, biphenyl, salicylate or toluene. Members of the genus *Rhodococcus* are known for their ubiquity in contaminated environments (Bell *et al*., 1998), which is explained by the genetic rearrangement and plasticity of the *Rhodococcus* genomes (Larkin *et al*., 2006). Other characteristics of this genus are its resistance to genetic manipulation (Larkin *et al*., 1998) and the redundancy of metabolic pathways and genes in their genomes (van der Geize and Dijkhuizen, 2004; McLeod *et al*., 2006), which complicates the elucidation of complete biodegradation pathways.

Naphthalene is a polycyclic aromatic hydrocarbon formed by two aromatic rings sharing two carbon atoms. It is one of the most common aromatic compounds present in the environment, and it is released as coal tar and coal tar products, such as creosote (Mueller *et al*., 1989). The genetics of naphthalene degradation pathways has been studied in detail in Gram-negative bacteria, such as *Pseudomonas* (Yen and Serdar, 1988) or *Ralstonia* species (Fuenmayor *et al*., 1998). In all known pathways, naphthalene catabolism starts with a dihydroxylation producing 1,2-dihydroxynaphthalene, followed by the cleavage of the hydroxylated aromatic ring, resulting in the formation of salicylate (upper pathway). Salicylate is further metabolized via catechol or gentisate pathways depending on the bacteria (lower pathway). In Pseudomonads, naphthalene degradation takes place via catechol formation (Eaton and Chapman, 1992), whereas in *Ralstonia* sp. strain U2 and *Polaromonas naphthalenivorans* CJ2, naphthalene catabolism follows the gentisate pathway (Fuenmayor *et al*., 1998; Jeon *et al*., 2006). Genetic organization is also different. In *Pseudomonas putida* G7, the best characterized naphthalene-degrading bacterium, *nah* genes are organized in two operons in plasmid pNAH7 (Schell *et al*., 1990). One operon codes for enzymes involved in the conversion of naphthalene to salicylate and the other encodes enzymes for the conversion of salicylate to pyruvate and acetyl coenzyme A via catechol production. In *Ralstonia* sp. strain U2, the *nag* genes are organized in a single operon coding for all enzymes involved in the conversion of naphthalene to pyruvate and fumarate through the gentisate pathway (Zhou *et al*., 2001). In both cases, transcription of *nag* and *nah* operons is controlled by LysR-type transcriptional regulators (Park *et al*., 2002a,b; Jones *et al*., 2003). These proteins act as positive regulators recognizing salicylate as the inducer molecule.

Although several *Rhodococcus* strains are known to grow on naphthalene, to our knowledge, a complete degradation pathway has not been characterized. Strains NCIMB 12038 (Boyd *et al*., 1997; Liu *et al*., 2011), B4 (Grund *et al*., 1992) and R7 (Di Gennaro *et al*., 2010) degrade naphthalene via gentisate, whereas strains P200 and P400 degrade it via catechol (Kulakova *et al*., 1996). Genes involved in naphthalene catabolism in these *Rhodococcus* strains are organized in different operons that are induced by naphthalene, salicylate or both (Kulakov *et al*., 2005; Di Gennaro *et al*., 2010; Liu *et al*., 2011).

In previous reports, combining proteomic, genetic and biochemical data, we have described the phthalate and tetralin degradation pathways in *Rhodococcus* sp. strain TFB (Tomás-Gallardo *et al*., 2006; 2009). Using the same approach, we show in this paper that naphthalene degradation in strain TFB seems to take place by combining, at least, three coordinated but independently regulated sets of genes.

## Results

### Proteome analysis of naphthalene TFB grown cells and identification of naphthalene-induced proteins

**Figure 1:**
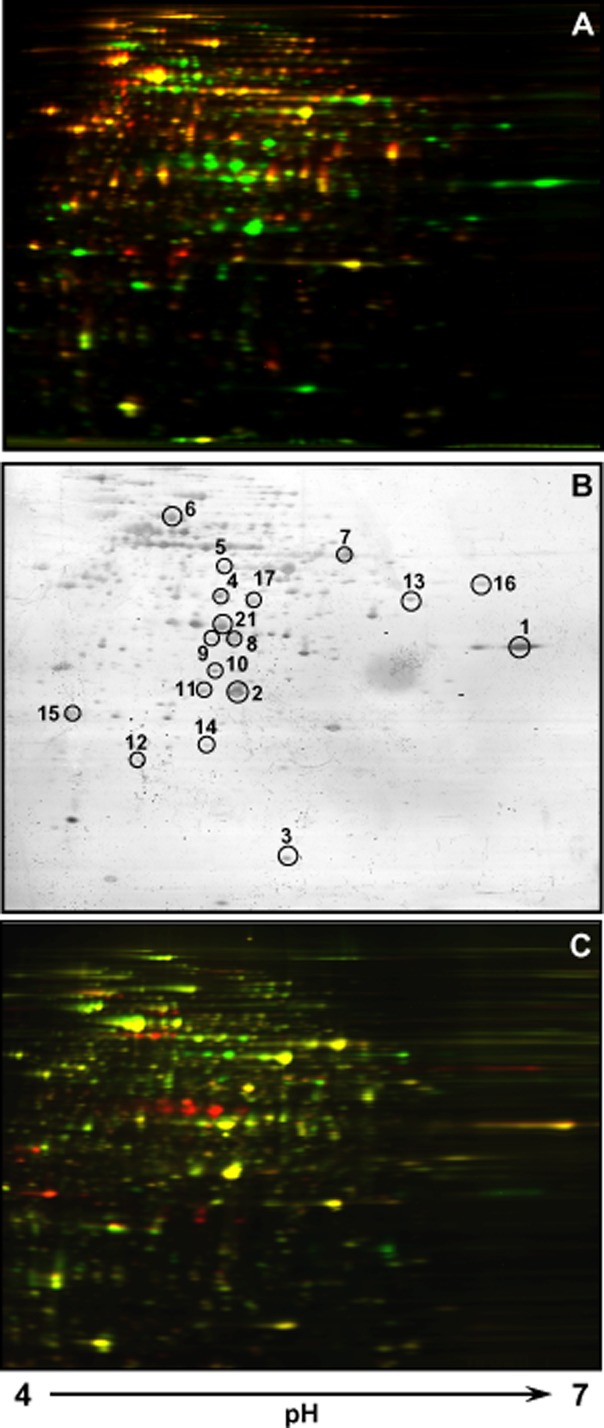
Proteomic analysis of TFB cells.A. Naphthalene-versus glucose-grown cells were compared using DIGE technology. Green spots are those specifically induced by naphthalene.B. Silver stained gel of naphthalene-versus glucose-grown cells used for DIGE analysis with the identified spots marked with a circle.C. Tetralin-versus naphthalene-grown cells using DIGE technology where yellow spots are those induced by both substrates, the green spots are induced only by tetralin, and the red spots are induced only by naphthalene.

Equal amounts of soluble protein extract from *Rhodococcus* sp. strain TFB grown exponentially on either glucose or naphthalene were labelled with Cy5 or Cy3 dyes respectively. Proteins specifically expressed in naphthalene-(green spots) or glucose-grown cells (red spots) were detected after scanning (Fig. 1A). A total of 103 (11.7%) out of 883 distinct spots showed naphthalene induction. Of the 103 spots, 49 could be individually selected from the two-dimensional differential in gel electrophoresis (2D-DIGE) gel (Fig. 1B) for analysis by mass spectrometry, which identified 16 different proteins (Table 1). Of these proteins, eight are also involved in the metabolism of tetralin in TFB (Tomás-Gallardo *et al*., 2009): the α and β subunits of the tetralin dioxygenase (ThnA1 and ThnA2; spots 7 and 12 respectively), the ferredoxin reductase (ThnA4, spot 5), the *cis*-biphenyl-2,3-dihydrodiol-2,3-dehydrogenase (ThnB, spots 2 and 11), a possible sterol transfer protein (ThnU, spot 3), the extradiol dioxygenase (ThnC, spot 8 and 9), the aldehyde dehydrogenase (ThnV, spot 4) and the β-ketoadipyl-CoA thiolase (ThnI, spot 13). We also found three proteins (spots 6, 10 and 17) previously identified as induced by tetralin in TFB proteome but not directly involved in tetralin biodegradation. Spot 6 is similar to a group of proteins with chaperone-like functions (Neuwald *et al*., 1999). Spot 10 was identified as an undecaprenyl pyrophosphate synthetase, an enzyme involved in cell wall synthesis. Induction of these spots might respond to the stress situation created when growing on naphthalene (Pumphrey and Madsen, 2007). Spot 17 was identified as a protein similar to a transposase from *Bacillus halodurans*.

**Table 1:** Naphthalene induced proteins identified by mass spectrometry

From gel in Fig. 1						
Spot	Accession code	Name and organism	Mw/kDa^a^	pI^a^	Peptides sequence^b^	Gene
1	gi|10567587	4-(2-oxocyclohexyl)-2-hydroxy-buta-2,4-dienoic acid hydrolase. *Sphingomonas macrogolitabida* strain TFA	32	6.11	CGHWAQLER LVNFYADPR	*thnD*
2, 11	gi|29647412	Cis-biphenyl-2,3-dihydrodiol-2,3-dehydrogenase. *Rhododococcus jostii* RHA1	28	4.95	LDTFVGNAAIWDFSTK TATGAIINCDGGMGVR	*thnB*
3	gi|111026222	Sterol transfer protein. *Rhodococcus jostii* RHA1	14	5.17	TPDFVLATK LDVWRQFADGKLRA	
4	gi|119718512	Acyl-CoA dehydrogenase domain-containing protein. *Nocardioides* sp. JS614	41.59	5.06	VMTLYEGTSQIQK	
5	gi|2072113	Ferredoxin reductase. *Escherichia coli* K12	43	5.6	HLPYERPPLSK	
6	gi|118470801	ATPase, AAA family protein. *Mycobacterium smegmatis* MC2 155.	65.3	4.85	SVLDTGAPGLR AIDTESNTGQYL IKIERPDAESAQDIFSK DFNSGAMIQNIVDR	
7	gi|111026201	Ethylbenzene dioxygenase alpha subunit. *Rhodococcus jostii* RHA1.	51	5.28	VFANSCPHR VCFADAGNR MMPVAQVASYK	*thnA1*
8, 9	gi|63148158	Catechol 2,3-dioxygenase. *Rhodococcus* sp. YU6	34	4.99	LLGLEGAVEYK GAVGTPVFMHCNNR DIFGHDNEVEGYGLDPIPLK	*thnC*
10	gi|16332030	Undecaprenyl pyrophosphate synthetase. *Synechocystis* sp.PCC 6803	28.8	6.6	QEIVHVCQAIAR	
12	gi|110825055	Ethylbenzene dioxygenase beta subunit. *Rhodococcus jostii* RHA1.	21	5.06	MAYYNDDLDMIFTR DEDRPLVGSREDTWR VYSNFFAFR	*thnA2*
13	gi|91787128	Beta-ketoadipyl CoA thiolase. *Polaromonas* sp. JS666	40.6	5.38	APFVFPK	
14	gi|226350019	Hypothetical protein ROP_pROB02–01880. *Rhodococcus opacus* B-4	18	5.2	LPAASATDLQR GDFVYTPPWIWHR	
15	gi|226361269	NAD(P)H-quinone oxidoreductase. *Rhodococcus opacus* B4	28.6	4.6	AALETAFAGVDK TLAVTGATGHLGR LVSGSEVGQR	
16	gi|85373346	Histidinol dehydrogenase. *Erythrobacter litoralis* HTCC2594	46.4	4.93	DVFDILARVK	
17	gi|10176610	Transposase. *Bacillus halodurans* C-125	50	8.9	YFAPTCVR	
21	gi|226361237	Putative heparin-binding hemagglutinin. *Rhodococcus opacus* B4	28.24	4.86	LSYAELR YQLDAAGVER FTADELR YELNAEMPR VASDLYTSLAER VLDLGDQAEEASKYQLDAAGVER EAAIQVSNVAIFNAATGK GATVELADGVEGYLR	
From gel in Fig. 2						
Spot	Accession code	Name and organism	Mw/kDa^a^	pI^a^	Peptides sequence^b^	Gene
11	gi|111020426	Flavin-binding monooxygenase *Rhodococcus jostii* RHA1	60.7	4.7	DITFDTR FAGQPEILR ANDTIADYIR GEVLLTGNWPR TPEFGGIDNFR KANDTIADYIR YFISGAGNLSVPK STPIDEITPTGVR ANDTIADYIRDR TFDERWNAGGFR ACMVYLGGAPTYR YLEHVADRFDLR GGLPLAEKWEHGPR SRYFISGAGNLSVPK YLEHVADRFDLRK LFIDSYQDILFDKK RPPLETNYYEA ATCDEVVAGGYSGFALTR TPEFGGIDNFRGEVLLTGNWPR NHFLGVPFNQVQPSALAVDAEER RPPLETNYYEAFNRDSVSVVDVK VAVIGTGASGIQAIPFIAEDAAELVVFQR TPNFATPLGNGPMDPNELADIKSNYADVR	
12	gi|111020425	Esterase/lipase *Rhodococcus jostii* RHA1	32.9	4.7	DEAEAYAESLR AVANGAGAIVVAATYRR VEDAHYESGGAQIALR AVVATFAGLQAPPEPVAR ALDAHAAELIAGLQAQGLK GQVLIYPVIDPNADLPSR AAGFEGLPPALVLTTENEVAR LRGQVLIYPVIDPNADLPSR	
13, 14,15,16,17		n.i.^c^				

a Mw and pI were calculated from the predicted protein sequences.

b Peptides subjected to tandem mass spectrometry (MS/MS) analysis are underlined.

c n.i., non-identified.

**Figure 2:**
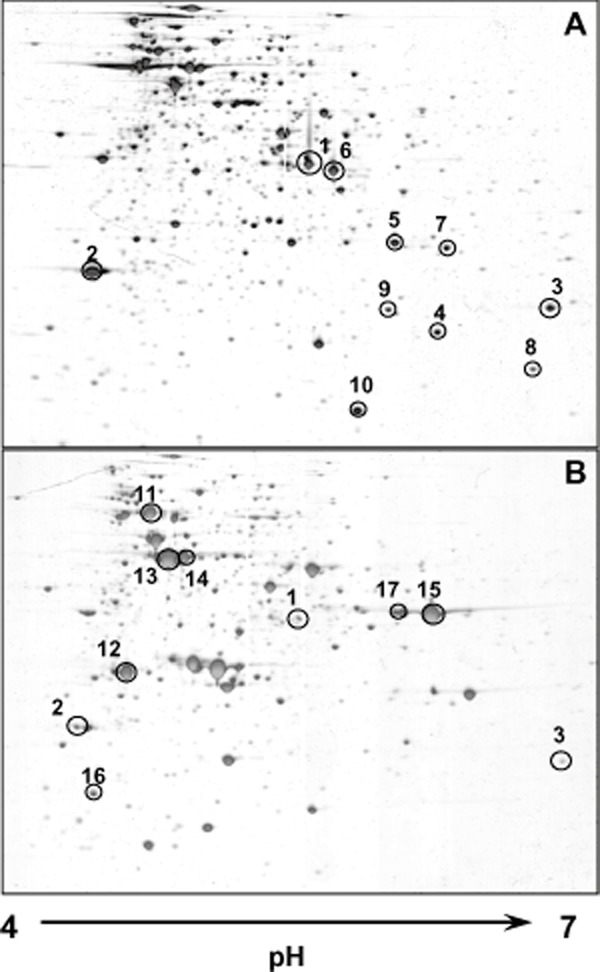
Proteomic analysis of TFB cells grown on salicylate (A) or naphthalene (B). Silver stained 2D gels where analysed with Image Master Platinum version 7.0 (GE Healthcare) and selected spots induced by each substrate were identified by matrix-assisted laser desorption/ionization mass spectrometry (MALDI-MS).

Spots 14, 15 and 21, present only on naphthalene cell extracts, were similar to proteins from *R. opacus* B4. Spot 14 was identified as an hypothetical protein with homology to the Cupin superfamily (Dunwell *et al*., 2004), and spot 15 was identified as a NAD(P)H-quinone oxidoreductase, an enzyme involved in cell detoxification in response a oxidative stress in different bacteria (Hong *et al*., 2008; Zhang *et al*., 2009). Spot 21 was identified as a possible heparin-binding hemaglutinin, an enzyme involved in mycobacterial aggregation (Menozzi *et al*., 1996).

To confirm that some of the identified proteins were induced in both tetralin-or naphthalene-grown cells, a 2D-DIGE analysis was performed using the soluble protein fraction from tetralin-grown cells labelled with Cy5 and the same fraction from naphthalene-grown cells labelled with Cy3 (Fig. 1C). As shown, most of the spots identified as naphthalene-induced proteins were also induced by tetralin (yellow spots), while few proteins are specifically induced by only one of the molecules.

### Analysis of key enzymatic activities on naphthalene degradation

Proteins similar to those known as involved in the production of salicylate from naphthalene were not identified by the proteomic analysis of the cells grown on naphthalene. To test whether salicylate was an intermediate in naphthalene metabolism in TFB, salicylaldehyde dehydrogenase activity was measured in cell-free extracts from TFB cells grown on glucose, tetralin, salicylate, naphthalene or glucose plus naphthalene. Salicylaldehyde-dependent NADH production was detected only in naphthalene-or naphthalene plus glucose-grown cells with similar activities (0.11 ± 0.04 U mg^−1^ protein and 0.14 ± 0.05 U mg^−1^ protein respectively). Induction the enzymatic activity that produces salicylate in the presence of naphthalene suggests that salicylate is an intermediate in naphthalene biodegradation. Interestingly, this activity is specifically induced by naphthalene but not by salicylate and is not subjected to catabolite repression by glucose.

To elucidate if salicylate is metabolized via catechol or gentisate, catechol 1,2-dioxygenase, catechol 2,3-dioxygenase and gentisate dioxygenase activities were assayed with cell-free extracts of TFB cells grown on glucose, naphthalene, salicylate and glucose plus salicylate. Catechol dioxygenase activity was undetectable in any condition (data not shown). However, gentisate dioxygenase activity was measured in naphthalene (0.2 ± 0.01 U mg^−1^ protein), salicylate (0.7 ± 0.21 U mg^−1^ protein) and glucose plus salicylate (1.2 ± 0.53 U mg^−1^ protein) TFB grown cells. This activity was 5.6-fold higher in salicylate-than in naphthalene-grown cells, thus indicating that salicylate is the real inducer. Besides, expression of its coding gene is not subjected to glucose-mediated catabolite repression as the activity found on salicylate plus glucose-grown cell extracts is even higher than that of salicylate-grown cells extracts.

### Proteomic analysis of TFB cells grown on salicylate

The different pattern of key enzymatic activities in salicylate-and naphthalene-grown cells suggested that several genes involved in naphthalene degradation could be induced by salicylate. A proteomic analysis was performed comparing the soluble proteome of TFB cells growing on each substrate. A total of 156 and 163 spots were detected in the naphthalene and salicylate proteomes respectively. After matching, 10 spots induced by salicylate were selected for identification by mass spectrometry. Three of the salicylate-induced spots (spots 1, 2 and 3) were also present in the naphthalene proteome although in lower quantity (Fig. 2), and seven were specific of salicylate-grown cells. Spots 1, 2 and 3 were identified as a gentisate 1,2-dioxygenase, a 3-maleylpyruvate isomerase and a mycothiol-dependent maleylpyruvate isomerase, respectively, very similar to those found in different *Rhodococcus* strains as involved in gentisate catabolism (Table 2). Further analysis of the 3-maleylpyruvate isomerase sequence revealed that this protein belongs to the fumaryl-acetoacetate hydrolases family, just like NagK, the fumaryl-pyruvate hydrolase involved in gentisate metabolism in *Ralstonia* sp. strain U2 (Zhou *et al*., 2001) and *Corynebacterium glutamicum* (Shen *et al*., 2005). Those three enzymes could be responsible for gentisate degradation in TFB following a micothiol-dependent pathway as it has been described in *R. jostii* RHA1 (Dosanjh *et al*., 2008), *Rhodococcus* sp strain NCIMB 12038 (Liu *et al*., 2011) and *Corynebacterium glutamicum* (Feng *et al*., 2006). No direct function on salicylate degradation could be assigned to the identified spots specifically induced by salicylate. Spot 4 is similar to a hypothetical protein of the Cupin superfamily different to the naphthalene-induced protein (spot 14) in the DIGE analysis but identified as a tetralin-induced protein in a previous analysis (Tomás-Gallardo *et al*., 2009). Spot 5 is similar to a 3-methyl-2-oxobutanoate hydroxymethyltransferase, a protein involved in biosynthesis of pantotenate and CoA. Its induction could be explained as a compensation for the inhibition of the activity by salicylate as it has been described in *Salmonella typhimurium* (Primerano and Burns, 1982). Spots 6 and 9 are proteins similar to an alanine dehydrogenase and a 3-hydroxyacyl-CoA dehydrogenase, respectively, both involved in central metabolism. Spot 7 was identified as an acyl-[acyl-carrier-protein] desaturase, an enzyme involved in fatty acid biosynthesis. Spot 8 was identified as a flavodoxin, an enzyme that catalyses electron transfer reactions. Spot 10 was not identified. Naphthalene-induced spots different from those selected in the 2D-DIGE analysis were sent for identification by mass spectrometry. Only two of seven spots were identified in this analysis (Fig. 2 and Table 1). Spots 11 and 12 are similar to a flavin-binding mono-oxygenase and an esterase/lipase, respectively, both located together in *R. jostii* RHA1 genome but with unknown functions. Spots 13, 14, 15, 16 and 17 could not be identified because their peptide mass fingerprinting did not match any protein in the databases.

**Table 2:** Identification of salicylate induced proteins by mass spectrometry

Spot	Accession code	Name and organism	Mw/kDa^a^	pI^a^	Peptides sequence	Gene
1	gi|111018863	Gentisate 1,2-dioxygenase *Rhodococcus jostii* RHA1	39.8	5.1	HAQNAFR AVPFVWK AGDLVPVGR TSSPIAAYR WEHTDAALR DVGSTVYQVFDGSGR YTNPTTGGDVMPTIR LWAHPGLRPLVGLDAK TSSPIAAYRWEHTDAALR	*nagI*
2	gi|58430768	3-Maleylpyruvate isomerase *Rhodococcus opacus* CIR2	29.8	4.5	TLQWDQGK DLPQYPTLFAK TLQWDQGKTFEK IICVGLNYANHIQEMGR KPGLYIQDGQTVEVTIEGLGTVR SIADNASGATVDLDGADYAPVVPHPGK LVEYISHIVTLQPGDVVITGTPGGVGHAR	*nagK*
3	gi|226360956	Mycothiol-dependent maleylpyruvate isomerase *Rhodococcus opacus* B4	25.9	5.2	LDEKWR GTAYFAQR VSGPLAAVVR RGTAYFAQR NLFDHTVAR TREVWIHAVDLGNGGR	*nagL*
4	gi|42475486	Hypothetical protein *Rhodococcus rhodochrous* K37	18.4	5.2	DMIVVPAGVPR HEDWDTLGFQAK AQIRYVGSGATGNHENDNR	
5	gi|111018182	3-Methyl-2-oxobutanoate hydroxymethyltransferase *Rhodococcus jostii* RHA1	30.4	5.4	FGNVGDELR RFGNVGDELR AGTFPAEEHSF LYGSAPSHDVPKR EGLAHAVKLEGGER WAMLTAYDYSSAR FMKEGLAHAVKLEGGER SDSKSSASTSEDRLYGSAPSHDVPKRK GAPHALVVADLPFGSYESSPEQALASATR	
6	gi|111021438	Alanine dehydrogenase *Rhodococcus jostii* RHA1	38.2	5.5	EIKNHEYR APTLVSNNLVSR VKEPIAEEYAR LAPQAGAYHLMR LRQDQVLFTYLHLAASK TTSIAYETVVGADGSLPLLAPMSEVAGR	
7	gi|111019251	Acyl-[acyl-carrier-protein]desaturase *Rhodococcus jostii* RHA1	35.8	5.6	DYLVVTR WNMFER QHLDDVVLPVLRK WTAEENKHSIVMR ELLHELEDVAEDNVNR AAMITNLLTEDNLPSYHR	
8	gi|111017066	Flavodoxin *Rhodococcus jostii* RHA1	19.1	5.6	ANGPALLTGR EWLDGLPPTHGK TLVVYESMFGNTR MRTLVVYESMFGNTR AGDEDLSGYDLVMVGGPTHVHGMSR	
9	gi|111020935	3-Hydroxyacyl-CoA dehydrogenase *Rhodococcus jostii* RHA1	25.7	5.4	TPLLGSLPEAAQQSLGGQVPHPSR	
10		n.i.^b^				

a Mw and pI were calculated from the predicted protein sequences.

b n.i., non-identified.

### Cloning and identification of *nag* genes of *Rhodococcus* sp. strain TFB

**Figure 3:**
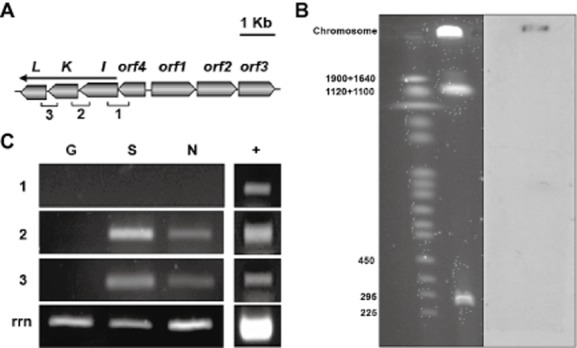
Genetic organization, chromosomal location and expression profile of *nag* genes in TFB.A. Genetic organization of *nag* genes: *nagI* (gentisate dioxygenase), *nagK* (fumarylpyruvate hydrolase) and *nagL* (mycothiol-dependent maleylpyruvate isomerase). Other *ORFs*: *orf1*, putative benzoate transporter; *orf2*, 3-hydroxybenzoate 6-monooxygenase; *orf3*, CorA-like putative magnesium transport protein; *orf4*, IclR-like regulatory protein.B. Genomic location of *nagI*. Southern blot hybridization showing the chromosomal location of *nag* genes. Lane 1, size markers. Lane 2, separated total TFB DNA after ethidium bromide staining. Lane 3, hybridization of Lane 2 with a *nagI* probe.C. Expression profile. RNA was isolated from mid-log glucose (G), salicylate (S) or naphthalene (N) grown cells. Intergenic amplified regions (1, 2 and 3) are shown with brackets. Amplification of 16S ribosomal RNA (rrn) was used as control of the amount of cDNA. Genomic DNA was used as template in control PCR (+).

A protein similar to the putative gentisate 1, 2-dioxygenase (NagI) encoded by the gene *ro01866* from *R. jostii* RHA1 was identified in the proteomic analysis. Based on its DNA sequence, primers FwGDORH and RvGDORH (Table 3) were designed to amplify an intragenic region. A 489 bp polymerase chain reaction (PCR) product, obtained using total DNA from *Rhodococcus* sp. strain TFB as template, was cloned and sequenced. Its nucleotide sequence shows 95% identity to the corresponding region of the *Rhodococcus* sp. RHA1 *ro01866* gene. The putative *nagI* PCR product was used to screen a TFB library, identifying a positive cosmid, named pMPO1109. The sequence of a 7192 bases fragment of pMPO1109 showed the presence of seven open reading frames (ORFs) (Fig. 3A), whose deduced amino acid sequences were screened against the peptide sequences obtained from the proteome analysis. Remarkably, peptides from spots 1, 2 and 3 perfectly matched sequences of the products of the ORFs named *nagI*, *nagK* and *nagL* respectively (Table 2). Upstream of the *nag* genes, there is an ORF encoding a protein similar to IclR-like transcriptional regulators (*orf4*). The most similar characterized protein is Ncg12921, an IclR type regulator involved in gentisate degradation regulation in *Corynebacterium* (Shen *et al*., 2005). Divergently transcribed to the IclR regulator gene, there are three more ORFs that seem to encode a benzoate transporter (*orf1*), a salicylate monooxygenase (*orf2*) and a CorA-like putative magnesium transport protein (*orf3*).

**Table 3:** Plasmids and oligonucleotides used in this work

Plasmid	Relevant characteristics	Reference
pBluescriptII SK+	Cloning vector, Ap^r^	Stratagene
pMPO1102	489 bp EcoRV PCR fragment with primers FwGDORH and RvGDORH cloned in pBluescriptII SK+	This work
pMPO1109	SuperCosI carring a 40 Kb fragment of TFB genomic DNA	This work
pMPO633	388 bp SalI-ScaI PCR fragment with primers thnA1ScaIPCRsolap and thnSA1SalIPCRsolap cloned in pMPO634	Tomás-Gallardo and colleagues (2009)
pMPO634	ScaI-XbaI PCR fragment containing *gfp* cloned in pNC9503	Tomás-Gallardo and colleagues (2009)
pNC9503	*E. coli*/*Rhodococcus* shuttle vector, Km^r^, thio^r^	H. Saeki, Japan Energy
Oligonucleotide	Target	Sequence
QRT-GDO Fw QRT-GDO Rv	*nagI*	ACGAACGGGATGTCGAGG ACGAAACCGATCAGCCGA
QRT-H Fw QRT-H Rv	*nagK*	TTGCCCTGATCCCACTGC TTACACGATGCGCGACTACC
QRT-MPI Fw QRT-MPI Rv	*nagL*	CAGGGTGCGCCACTTCTC AACCTGTTCGATCACACCGTC
QRT-ICLR Fw QRT-ICLR Rv	*nagR*	TGGTACATGCGTTTCTCGTCC GATGCTGGTGTACCGAGGGTT
QRT-SMO Fw QRT-SMO Rv	*nagG*	GCGGTGAGATGCTCAACCA CCTTGTAACCGGGCGATTC
QRT-MFS Fw QRT-MFS Rv	*nagU*	GCTGGTTTACGTCATCTTCGTG TGAGTTCCGTGTGTCCCGA
QPCR-ThnSFw QPCR-ThnSRev	*thnS*	GCGAATGTCGAGACCAGTTATG ACCCCCACTATGTCGATCTCAC
QRT thnDFw QRT thnDRv	*thnD*	CCTCTCGCTCAACGAGGAAC AAATCACGGGCGAGCTTCT
QRT thnA1Fw QRT thnA1Rv	*thnA1*	ATATGGGTGAGGATGCGGTG GAACGGAGTTGTCCCGGTG
thnSRT-PCRFw thnSRT-PCRRev	*thnS*	CAGCGAACACGCCTAGAGG GACGACGCTGGTCACATCG
RTorf7-fw 3-624rev	*thnU-A4*	CGACATCAAGGAGGGAAT CTGGTCACGGAGAGCGG
RT-ExDio RT-Aldh	*thnC-G*	GGATGGCTGTGGGAACCAG TGCGAACGTGATCGGTGTC

Because the naphthalene and tetralin-induced *thn* genes of TFB are located in the linear mega-plasmid pTFB1 of approximately 1.1 Mb (Tomás-Gallardo *et al*., 2009), we decided to analyse the location of *nag* genes using the 489 bp internal *nagI* fragment as the probe in Southern blotting experiments with TFB total DNA separated by pulse field electrophoresis gel. The two large plasmids that TFB bears were easily detected as previously reported, but none of them hybridized to the *nagI* probe (Fig. 3B). As described for *R. jostii* RHA1, we found that the *nag* genes are located on the TFB chromosome (McLeod *et al*., 2006).

### Transcriptional characterization of *thn*, *nag*, *orf1*, *orf2*, *orf3* and *orf4* genes

In order to characterize the expression pattern of some of the identified genes and their transcriptional organization, retrotranscription (RT)-PCR and quantitative real-time PCR (qPCR) analyses were carried out.

For the determination of the transcriptional units of *thn* and *nag* genes, RT-PCR assays were carried out using RNA obtained from glucose-, naphthalene-or salicylate-grown cells using the oligonucleotides described in Table 3. Intergenic regions corresponding to *nag* genes were detected in both naphthalene and salicylate growing cells (Fig. 3C). These results suggest that *nagIKL* genes are transcribed as an operon, while *orf4* is constitutively transcribed as an independent and low expressed cistron. PCR products of the expected sizes for each intergenic region of the *thn* genes were detected in naphthalene-but not salicylate-growing cells (not shown), and indicated that the transcriptional units for the *thn* genes are the same as those described when using tetralin as the inducer (Tomás-Gallardo *et al*., 2009).

For qPCR, total RNA isolated from TFB cells grown to mid-log phase with glucose, naphthalene, salicylate, and naphthalene or salicylate plus 2 h in the presence of glucose was converted to cDNA as described in Materials and Methods. As shown in Table 4, catabolic *thnA1* and *thnD* genes, and the regulatory *thnS* gene are induced by naphthalene but not by salicylate. Besides, the presence of glucose provokes a partial catabolite repression of these *thn* genes (2.4-fold for *thnS* and 3.6-fold for *thnA1* and *thnD*). On the contrary, the highest induction of the *nagIKL* genes is achieved in the presence of salicylate. A lower level of induction is also detected when naphthalene is the carbon source, but this induction may be due to the salicylate produced during naphthalene degradation. Intriguingly, *nagIKL* expression is not subjected to glucose-mediated catabolite repression at all when induced by salicylate, while it is fully repressed when induced by naphthalene. However, this difference may be explained by an indirect effect of the carbon catabolite repression of *thn* genes, which prevents conversion of naphthalene into the real inducer salicylate. Expression of *orf1*, *orf2, orf3* and *orf4* genes was not detected in any condition (not shown).

**Table 4:** qPCR analysis of the *thn* and *nag* genes expression in different growth conditions

	*thnA1*	*thnD*	*thnS*	*nagI*	*nagK*	*nagL*
Glucose	ND	ND	0.22 ± 0.04	ND	ND	ND
Salicylate	ND	ND	ND	6.41 ± 0.16	5.37 ± 0.44	1.61 ± 0.24
Salicylate plus glucose	ND	ND	ND	5.92 ± 0.39	4.62 ± 2.88	2.03 ± 0.38
Naphtalene	2.98 ± 0.91	2.63 ± 0.56	1.63 ± 0.49	1.4 ± 0.14	0.19 ± 0.02	0.24 ± 0.05
Naphthalene plus glucose	0.81 ± 0.49	0.74 ± 0.54	0.69 ± 0.07	ND	ND	ND

Numbers represent quantities in arbitrary units calculated based on a DNA calibration curve. Values are the means ± standard deviations of at least three independent experiments.

ND, not detected.

### Inducer profile of TFB *thn* genes

**Figure 4:**
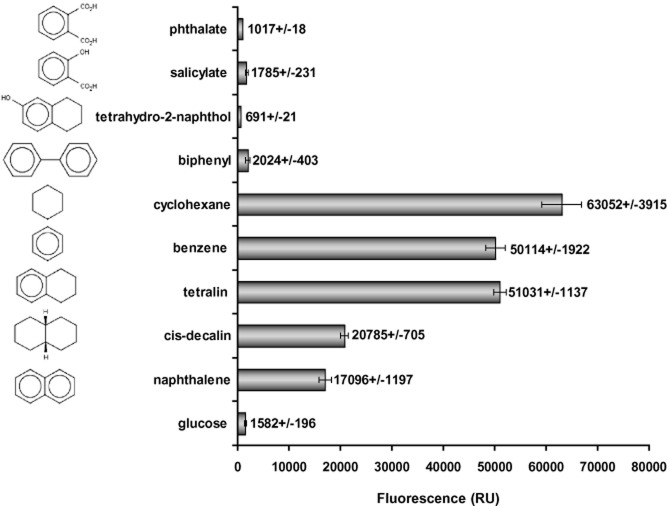
Inducer profile of TFB *thn* genes. Fluorescence of TFB cells carrying plasmid pMPO633 (with a *gfp::thnA1* translational fusion) in the presence of each compound was measured in relative units (RUs).

Taking in consideration that the expression of *thn* genes is a prerequisite for naphthalene degradation in TFB, we performed a more complete analysis of the induction profile of TFB *thn* genes that was carried out by examining expression of a *thnA1::gfp* translational fusion in the presence of molecules that may share some structural or functional features with tetralin and/or naphthalene. As shown in Fig. 4, cyclohexane and benzene (monocyclic molecules) induce *thn* gene expression at similar levels as tetralin. In addition, naphthalene and *cis*-decalin, molecules formed by two aromatic or aliphatic rings, respectively, sharing two carbon atoms, also induce *thn* genes, but in a less efficient mode (33% and 40% of the levels obtained with tetralin respectively). On the other hand, monocyclic (phthalate and salicylate) or bicyclic (5,6,7,8-tetrahydro-2-naphtol and biphenyl) molecules containing substitutions do not induce *thn* genes at all.

## Discussion

Using proteomic and genetic approaches, we show in this work that TFB naphthalene degradation ability may be the result of the combination of different catabolic pathways. Genes encoding the enzymes of these pathways are located in different replicons, have their own specific induction profile and respond differently to the presence of an alternative carbon source.

**Figure 5:**
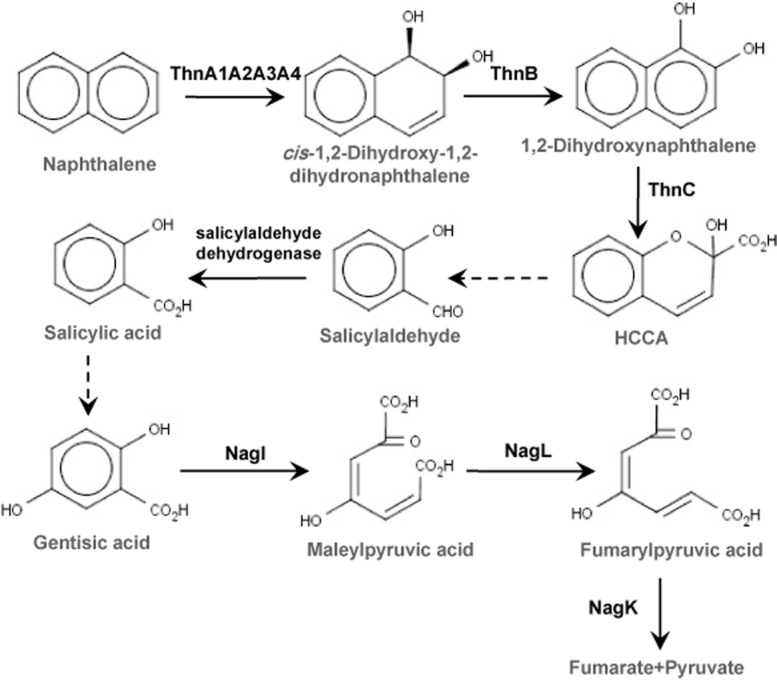
Naphthalene degradation pathway proposed in *Rhodococcus* sp. strain TFB. Reactions catalysed by unknown enzymes are shown with dotted lines. HCCA, 2-hydroxycromene-2-carboxylate.

In TFB, naphthalene induces the tetralin degradation enzymes, as happens in *Sphingomonas macrogolitabida* strain TFA, an α-proteobacterium whose tetralin degradation pathway and *thn* genes have been thoroughly characterized. In TFA, naphthalene can be initially transformed (although it is not able to grow on naphthalene), and the specific activity of TFA-ThnC (the extradiol dioxygenase), which is 65% identical to TFB-ThnC, is 1.6-fold higher when using 1,2 dihydroxynaphthalene as the substrate than that using the corresponding tetralin intermediate (Andújar *et al*., 2000). Because Thn proteins are the most prominently induced in naphthalene-gown cells, we propose that the ThnA1A2A3A4 (the initial dioxygenase complex), ThnB (dehydrogenase) and ThnC enzymes, which catalyse the first three steps of the tetralin degradation pathway, may also convert naphthalene into 2-hydroxychromene-2-carboxylate (HCCA) in TFB (Fig. 5). In TFB, both, the *thn* structural and the *thn* regulatory genes (a two-component system formed by ThnST) are located at the pTFB1 mega-plasmid and are subjected to catabolite repression (Table 4). Recently, a cyclic AMP receptor protein (CRP)-like protein has been identified as involved in the repression of the *thn* genes in the presence of glucose (Tomás-Gallardo *et al*., 2012). In TFB, *thn* genes are also induced by compounds that can hardly be considered as substrates of the encoded enzymes (i.e. *cis*-decalin or cyclohexane, Fig. 4). On the other hand, TFB-*thn* genes are not induced by compounds like biphenyl, which is a good substrate of TFB-ThnD (Tomás-Gallardo *et al*., 2009). This proves the laxity of the regulatory system in recognizing putative inducers. A broad spectrum of inducers provokes the gratuitous induction of the pathway but can confer an advantage in the degradation of new molecules. Besides, in contrast with the regulation of naphthalene degradation in *Pseudomonas*, neither TFB-nor TFA-*thn* genes are induced by salicylate (López-Sánchez, 2009; Table 4).

In pseudomonads, HCCA is converted to salicylate in three steps that involve an isomerase, a hydratase-aldolase (named NarC in *Rhodococcus* or NahE in *Pseudomonas*) that yields pyruvate and salicylaldehyde, and a salicylaldehyde dehydrogenase that converts the latter into salicylate. We were not able to identify these enzymes or the encoding genes either by proteomics or by PCR using degenerate primers (not shown). The isomerase activity has been shown not to be essential for growth on naphthalene (Royle *et al*., 1981; Eaton and Chapman, 1992) and has not been identified in other naphthalene degrading rhodococci. Therefore, it is possible that TFB lacked this enzyme, which could explain its high doubling time (35 h; Tomás-Gallardo *et al*., 2006) when growing with naphthalene as carbon and energy source. In both *R. opacus* R7 (Di Gennaro *et al*., 2010) and *Rhodococcus* sp. NCIMB12038 (Kulakov *et al*., 2005), the *narC* encoding the hydratase-aldolase is not located in the vicinity of the *nar* genes, and the same could be happening in TFB. However, the similarity of the TFB *narC* to other rhodococcal *narC* genes seems not to be high enough to allow its detection by PCR. A less likely possibility is that ThnE and ThnF, the hydratase and aldolase of the tetralin degradation pathway, could use naphthalene intermediates as substrates. Another possibility is that the naphthalene-induced spot identified as an esterase/lipase (spot 12, Fig. 2 and Table 1), which according to Pfam carries an alpha/beta hydrolase domain, was involved in this conversion by an unknown mechanism. Functional characterization of this enzyme would be required to confirm this hypothesis.

We have detected a salicylaldehyde dehydrogenase activity in naphthalene-grown cells that is undetectable in salicylate-grown cells. This indicates that the regulation of the upper pathway (conversion of naphthalene into salicylate) is different in rhodococci as compared with pseudomonads, in which salicylate induces both the upper and the lower pathway (conversion of salicylate into central intermediates) (Yen and Serdar, 1988). Besides, this activity in TFB is neither induced by tetralin nor shows glucose catabolite repression when induced by naphthalene, thus indicating that its encoding gene, which remains unidentified, does not belong to the *thn* genes regulon, which showed a partial but obvious catabolite repression by glucose (Table 4).

Two different pathways to convert salicylate into gentisate have been described in the literature, one via a salicylate-5-hydroxylase activity that involves two structural subunits that form a salicylate-5-hydroxylase linked to an electron transport chain (Suemori *et al*., 1993; Fuenmayor *et al*., 1998) and the other via a CoA-dependent pathway as it has been proposed in *Streptomyces* sp strain WA46 (Ishiyama *et al*., 2004) and *R. opacus* R7 (Di Gennaro *et al*., 2010). In TFB, only a protein similar to a flavin-binding monooxygenase in *R. jostii* RHA1 has been identified as induced by naphthalene (spot 11, Fig. 2 and Table 1), but its role in the catabolism of this compound remains unclear as no salicylate-5-hydroxylase activity nor the CoA-dependent pathway have been detected in TFB cell extracts (not shown).

Gentisate degradation seems to take place in TFB as it has been described in other rhodococci (Liu *et al*., 2011) as the TFB *nag* genes are very similar (more than 90% identical) to other rhodococcal genes found in RHA1, *R. opacus* B4, or *Rhodococcus* sp. NCIMB 12038. Proteomic, gene expression and gentisate dioxygenase activity measurement indicate that TFB *nag* genes are specifically induced by salicylate and not by naphthalene, and their products are involved in gentisate metabolism. Upstream of *nagI* and orf4, a palindromic region (TTCTGCACAGCAGAA and TTCTGCTGTGCAGAA respectively), also found upstream of the *R. opacus* B4 and RHA1 gentisate dioxygenase genes (ROP_15440 and RHA1_ro01866 respectively), could be recognized by the IclR-like putative regulator of these genes (the *orf4* product). This type of transcriptional regulators can act as activators or repressors and there is not a clear consensus sequence for their DNA-binding sites (Molina-Henares *et al*., 2005).

Although further experiments should be carried out to completely identify each enzyme and reaction involved in conversion of HCCA into salicylaldehyde and salicylate into gentisate, the data presented in this paper provide evidences supporting a biodegradation pathway for naphthalene in TFB (Fig. 5) in which at least three sets of enzymes, differently regulated, participate: the Thn enzymes induced by naphthalene or tetralin but not by salicylate, the salicylaldehyde dehydrogenase induced by naphthalene but not by salicylate or tetralin, and the Nag enzymes induced by salicylate but not by naphthalene or tetralin. Therefore, it represents a paradigmatic example of how a biodegradation pathway can be assembled using genes belonging to different regulons.

## Experimental procedures

### Bacterial strains, culture conditions, plasmids and primers

*Rhodococcus* sp. strain TFB (Tomás-Gallardo *et al*., 2006) was grown on minimal medium (MM) (Dorn *et al*., 1974) at 30°C with tetralin supplied in gas phase, naphthalene supplied as solid phase, 12 mM of sodium salicylate or 12 mM of glucose as carbon and energy sources. Lysogeny broth (LB; Bertani, 2004) was used as rich media. *Escherichia coli* strains were grown in LB medium containing the appropriate antibiotics. *Escherichia coli* DH5α (Hanahan, 1983) was used for DNA manipulation.

For carbon catabolite repression assays, cells were grown on MM with glucose until optical density (OD)_600_ = 1, diluted 10-fold on MM with naphthalene plus glucose or salicylate plus glucose. After incubation for 12 h, cultures were diluted fivefold in the same medium and incubated until OD_600_ = 1 was reached.

Plasmids and primers used in this work are listed in Table 3.

### Enzyme assays

Cell-free extracts were prepared by cell disruption using a French press. Exponentially growing cells (OD_600_ = 1) on MM supplemented with glucose, tetralin, naphthalene, salicylate, glucose plus naphthalene or glucose plus salicylate were harvested, washed once with phosphate buffer (17 mM Na_2_HPO_4_, 7.35 mM KH_2_PO_4_, pH 7.2), resuspended in 2 ml of phosphate buffer and disrupted. Cell extracts were centrifuged at 10,000 *g* for 20 min at 4°C, and the supernatants were collected for further assays.

Salicylaldehyde dehydrogenase activity was performed as described by Shamsuzzaman and Barnsley (1974) with several modifications. The reaction was determined from the rate of increase of absorbance at 340 nm because of the formation of NADH. The reaction started by adding 20 μl of fresh cell extract (50–250 μg soluble protein) to a 980 μl of reaction mixture containing 20 mM tetrasodium pyrophosphate buffer HCl pH 8.5, 0.1 mM salicylaldehyde and 10 mM NAD^+^. Production of NADH was calculated using an extinction coefficient of 3.84 mM^−1^ cm^−1^. One enzyme unit (U) was defined as the amount of enzyme that catalyses the production of 1 μmol of NADH per minute.

Gentisate dioxygenase activity was performed as described by Adams and colleagues (2006) with several modifications. The reaction started with the addition of 20 μl of cell extract (50–250 μg soluble protein) to the reaction mixture containing 979 μl phosphate buffer (described earlier) and 1 μl sodium gentisate 0.25 M. The activity was determined by measuring the increase of absorbance at 330 nm. The activity was calculated using an extinction coefficient for maleylpyruvate of 10.8 mM^−1^ cm^−1^. One enzyme unit (U) was defined as the amount of enzyme that catalyses the production of 1 μmol of maleylpyruvate per minute.

### Protein sample preparation, fluorophore labelling and 2D electrophoresis analysis

TFB cell free extracts, fluorophores labelling, 2D electrophoresis and protein identification were carried out as described in Tomás-Gallardo and colleagues (2006). For DIGE analysis, fluorescent gel images were obtained by scanning using a Typhoon TM 9400 Scanner and analysed with DeCyder Differential Analysis software version 5.01 (GE Healthcare, GE Healthcare Europe GmbH, Freiburg, Germany). Proteins selected for identification where those with an expression ratio level higher than 1.5 and a *t*-test lower than 0.05. For standard 2D analysis, silver-stained gel images were obtained by scanning using ImageScanner III software (GE Healthcare) and analysed with ImageMaster Platinum software version 7.0 (GE Healthcare). Theoretical molecular masses and isoelectric points of the proteins of interest were calculated using EXPASY tools. Identification of the selected spots was carried out by mass spectrometry at the Proteomic Unit of Centro Nacional de Investigaciones Cardiovasculares in Madrid, Spain.

### Total RNA sample preparation, RT-PCR and qPCR assays

Total RNA of *Rhodococcus* sp. strain TFB was isolated following the method described in Tomás-Gallardo and colleagues (2006). PCR amplification of 16S rDNA and some *thn* genes with RNA samples as template was conducted to test for DNA contamination. DNA-free RNA samples (2 μg) were converted to cDNA using the High Capacity cDNA Reserve Transcription kit (Applied Biosystems, Alcobendas, Madrid, Spain) following the manufacturer's instructions. cDNA was cleaned using the QIAquick® PCR Purification kit (Qiagen, Limburg, Netherlands). Equal amounts of cDNA were used as templates in PCR reactions with primers listed in Table 3. Semiquantitative PCR reactions were performed with 30 ng of cDNA using the puReTaq Ready-To-Go PCR beads (GE Healthcare) and consisted of 20 cycles of 30 s at 94°C, 30 s at 59°C and 45 s at 72°C. As loading control, primers f27 and r519 amplifiying 16S gene (Hugenholtz *et al*., 1998) were used with 20 pmol of cDNA.

qPCR was performed using SYBR Green technology in an ABI Prism 7000 Sequence Detection System (Applied Biosystems) following the method described previously by Yuste and colleagues (2006). Samples were initially denatured by heating at 95°C for 10 min. A 40-cycle amplification and quantification programme was followed (95°C for 15 s and 60°C for 1 min, with a single fluorescence measurement per cycle according to manufacturers' recommendations). A final extension cycle (72°C, 1 min) was performed. cDNAs (10 ng) from three biological samples of each condition were amplified in quadruplicate in separate PCR reactions using 0.3 mM of each primer. All PCR products were between 50 and 100 bp in length. Standard curves were made using serial dilutions from 50 to 0.01 ng of TFB genomic DNA to quantify the relative abundance of transcripts. Primers used are described on Table 3. Value of quantification (Qt) is expressed as quantity of transcript relative to the standard curve.

### Screening for genes encoding gentisate dioxygenases

A total TFB DNA library was screened by colony hybridization using a probe targeting gentisate dioxygenase gene. The probe was obtained by PCR using primers FwGDORH and RvGDORH (Table 3), whose design was based on the peptide sequence obtained from the proteomic analysis and the nucleotide sequence of *ro01866* from *R. jostii* RHA1 that encodes a putative gentisate dioxygenase. PCR was carried out using 50 ng of total DNA as template and the puRe-Taq Ready-To-Go PCR Beads (GE Healthcare) under the following amplification conditions: 95°C for 10 min, followed by 30 cycles of 95°C for 30 s, 65°C for 30 s and 72°C for 30 s. The 489 bp fragment was cloned into EcoRV-cleaved pBluescript II SK+ (Stratagene, Santa Clara, California, USA) to obtain pMPO1102. This fragment was used to probe a TFB library by Southern analysis (Sambrook *et al*., 1990) to get cosmid pMPO1109. A 7192 bp fragment from pMPO1109 was commercially sequenced.

The obtained DNA sequence was compared with those in the databases using BLASTN 2.2.6 (Altschul *et al*., 1997) and submitted to GenBank under Accession Number JF754465.

### Plasmid isolation, pulsed field gel electrophoresis, linearity determination and Southern blot hybridization

For detection of extra chromosomal DNA, plasmid isolation and pulse field gel electrophoresis assays were developed as described by König and colleagues (2004). Yeast Chromosome PFG marker (New England Biolabs, IZASA, Barcelona, Spain) was used as reference. After electrophoresis, DNA was transferred to Hybond-N+ nylon membranes (GE Healthcare) and subjected to Southern hybridization with a *nagI* probe.

### Fluorescence measurements of *gfp* fusions

Fluorescence of exponentially grown TFB cells harbouring the plasmid pMPO633 was measured using a POLARstar Omega Fluorimeter (BMG Labtech, Ortenberg, Germany) with the EM520 emission filter, the 485BP1 excitation filter and an adjusted gain of 1000. The fluorescence measurement was normalized using the OD_600_ of each culture. Tetralin, naphthalene, cyclohexane, *cis*-decalin and benzene were supplied in the gas phase. Soluble compounds were added at the following concentrations: 2 mM salicylate, 2 mM benzoate, 0.5 mM tretrahydro-2-naphthol, 0.5 mM biphenyl, 12 mM glucose and 12 mM phthalate.

## References

[b1] Adams MA, Singh VK, Keller BO, Jia Z (2006). Structural and biochemical characterization of gentisate 1,2-dioxygenase from *Escherichia coli* O157:H7. Mol Microbiol.

[b2] Altschul SF, Madden TL, Schaffer AA, Zhang J, Zhang Z, Miller W, Lipman DJ (1997). Gapped BLAST and PSI-BLAST: a new generation of protein database search programs. Nucleic Acids Res.

[b3] Andújar E, Hernáez MJ, Kaschabek SR, Reineke W, Santero E (2000). Identification of an extradiol dioxygenase involved in tetralin biodegradation: gene sequence analysis and purification and characterization of the gene product. J Bacteriol.

[b4] Bell KS, Philp JC, Aw DW, Christofi N (1998). The genus Rhodococcus. J Appl Microbiol.

[b5] Bertani G (2004). Lysogeny at mid-twentieth century: P1, P2, and other experimental systems. J Bacteriol.

[b6] Boyd C, Larkin MJ, Reid KA, Sharma ND, Wilson K (1997). Metabolism of naphthalene, 1-naphthol, indene, and indole by *Rhodococcus* sp. strain NCIMB 12038. Appl Environ Microbiol.

[b7] Di Gennaro P, Terreni P, Masi G, Botti S, De Ferra F, Bestetti G (2010). Identification and characterization of genes involved in naphthalene degradation in *Rhodococcus opacus* R7. Appl Microbiol Biotechnol.

[b8] Dorn E, Hellwig M, Reineke W, Knackmuss HJ (1974). Isolation and characterization of a 3-chlorobenzoate degrading pseudomonad. Arch Microbiol.

[b9] Dosanjh M, Newton GL, Davies J (2008). Characterization of a mycothiol ligase mutant of *Rhodococcus jostii* RHA1. Res Microbiol.

[b11] Dunwell JM, Purvis A, Khuri S (2004). Cupins: the most functionally diverse protein superfamily?. Phytochemistry.

[b12] Eaton RW, Chapman PJ (1992). Bacterial metabolism of naphthalene: construction and use of recombinant bacteria to study ring cleavage of 1,2-dihydroxynaphthalene and subsequent reactions. J Bacteriol.

[b13] Feng J, Che Y, Milse J, Yin YJ, Liu L, Rückert C (2006). The gene *ncgl2918* encodes a novel maleylpyruvate isomerase that needs mycothiol as cofactor and links mycothiol biosynthesis and gentisate assimilation in *Corynebacterium glutamicum*. J Biol Chem.

[b14] Fuenmayor SL, Wild M, Boyes AL, Williams PA (1998). A gene cluster encoding steps in conversion of naphthalene to gentisate in *Pseudomonas* sp. strain U2. J Bacteriol.

[b15] van der Geize R, Dijkhuizen L (2004). Harnessing the catabolic diversity of rhodococci for environmental and biotechnological applications. Curr Opin Microbiol.

[b16] Grund E, Denecke B, Eichenlaub R (1992). Naphthalene degradation via salicylate and gentisate by *Rhodococcus* sp. strain B4. Appl Environ Microbiol.

[b17] Hanahan D (1983). Studies on transformation of *Escherichia coli* with plasmids. J Mol Biol.

[b18] Hong Y, Wang G, Maier RJ (2008). The NADPH quinone reductase MdaB confers oxidative stress resistance to *Helicobacter hepaticus*. Microb Pathog.

[b19] Hugenholtz P, Pitulle C, Hershberger KL, Pace NR (1998). Novel division level bacterial diversity in a Yellowstone hot spring. J Bacteriol.

[b20] Ishiyama D, Vujaklija D, Davies J (2004). Novel pathway of salicylate degradation by *Streptomyces* sp. strain WA46. Appl Environ Microbiol.

[b21] Jeon CO, Park M, Ro HS, Park W, Madsen EL (2006). The naphthalene catabolic (nag) genes of *Polaromonas naphthalenivorans* CJ2: evolutionary implications for two gene clusters and novel regulatory control. Appl Environ Microbiol.

[b22] Jones RM, Britt-Compton B, Williams PA (2003). The naphthalene catabolic (*nag*) genes of *Ralstonia* sp. strain U2 are an operon that is regulated by NagR, a LysR-type transcriptional regulator. J Bacteriol.

[b23] König C, Eulberg D, Gröning J, Lakner S, Seibert V, Kaschabek SR, Schlömann M (2004). A linear megaplasmid, p1CP, carrying the genes for chlorocatechol catabolism of *Rhodococcus opacus* 1CP. Microbiology.

[b24] Kulakov LA, Chen S, Allen CC, Larkin MJ (2005). Web-type evolution of *Rhodococcus* gene clusters associated with utilization of naphthalene. Appl Environ Microbiol.

[b25] Kulakova AN, Reid KA, Larkin MJ, Allen CC, Kulakov LA (1996). Isolation of *Rhodococcus rhodochrous* NCIMB13064 derivatives with new biodegradative abilities. FEMS Microbiol Lett.

[b26] Larkin MJ, De Mot R, Kulakov LA, Nagy I (1998). Applied aspects of *Rhodococcus* genetics. Antonie Van Leeuwenhoek.

[b27] Larkin MJ, Kulakov LA, Allen CC (2006). Biodegradation by members of the genus *Rhodococcus*: biochemistry, physiology, and genetic adaptation. Adv Appl Microbiol.

[b28] Liu TT, Xu Y, Liu H, Luo S, Yin YJ, Liu SJ, Zhou NY (2011). Functional characterization of a gene cluster involved in gentisate catabolism in *Rhodococcus* sp. strain NCIMB 12038. Appl Microbiol Biotechnol.

[b29] López-Sánchez A (2009).

[b30] McLeod MP, Warren RL, Hsiao WW, Araki N, Myhre M, Fernandes C (2006). The complete genome of *Rhodococcus* sp. RHA1 provides insights into a catabolic powerhouse. Proc Natl Acad Sci USA.

[b31] Menozzi FD, Rouse JH, Alavi M, Laude-Sharp M, Muller J, Bischoff R (1996). Identification of a heparin-binding hemagglutinin present in mycobacteria. J Exp Med.

[b32] Molina-Henares AJ, Krell T, Guazzaroni ME, Segura A, Ramos JL (2005). Members of the IclR family of bacterial transcriptional regulators function as activators and/or repressors. FEMS Microbiol Rev.

[b33] Mueller JG, Chapman PJ, Pritchard PH (1989). Action of a fluoranthene-utilizing bacterial community on polycyclic aromatic hydrocarbon components of creosote. Appl Environ Microbiol.

[b34] Neuwald AF, Aravind L, Spouge JL, Koonin EV (1999). AAA+: a class of chaperone-like ATPases associated with the assembly, operation, and disassembly of protein complexes. Genome Res.

[b35] Park W, Jeon CO, Madsen EL (2002a). Interaction of NahR, a LysR-type transcriptional regulator, with the alpha subunit of RNA polymerase in the naphthalene degrading bacterium, *Pseudomonas putida* NCIB 9816-4. FEMS Microbiol Lett.

[b36] Park W, Padmanabhan P, Padmanabhan S, Zylstra GJ, Madsen EL (2002b). *nahR*, encoding a LysR-type transcriptional regulator, is highly conserved among naphthalene-degrading bacteria isolated from a coal tar waste-contaminated site and in extracted community DNA. Microbiology.

[b37] Primerano DA, Burns RO (1982). Metabolic basis for the isoleucine, pantothenate or methionine requirement of ilvG strains of *Salmonella typhimurium*. J Bacteriol.

[b38] Pumphrey GM, Madsen EL (2007). Naphthalene metabolism and growth inhibition by naphthalene in *Polaromonas naphthalenivorans* strain CJ2. Microbiology.

[b39] Royle PL, Matsumoto H, Holloway BW (1981). Genetic circularity of the *Pseudomonas aeruginosa* PAO chromosome. J Bacteriol.

[b40] Sambrook J, Fritsch EF, Maniatis T

[b41] Schell MA, Brown PH, Raju S (1990). Use of saturation mutagenesis to localize probable functional domains in the NahR protein, a LysR-type transcription activator. J Biol Chem.

[b42] Shamsuzzaman KM, Barnsley EA (1974). The regulation of naphthalene metabolism in Pseudomonads. Biochem Biophys Res Commun.

[b43] Shen XH, Jiang CY, Huang Y, Liu ZP, Liu SJ (2005). Functional identification of novel genes involved in the glutathione-independent gentisate pathway in *Corynebacterium glutamicum*. Appl Environ Microbiol.

[b44] Suemori A, Kurane R, Tomizuka N (1993). Purification and properties of 3 types of monohydroxybenzoate oxygenase from *Rhodococcus erythropolis* S-1. Biosci Biotechnol Biochem.

[b45] Tomás-Gallardo L, Canosa I, Santero E, Camafeita E, Calvo E, López JA, Floriano B (2006). Proteomic and transcriptional characterization of aromatic degradation pathways in *Rhodoccocus* sp. strain TFB. Proteomics.

[b46] Tomás-Gallardo L, Santero E, Camafeita E, Calvo E, Schlömann M, Floriano B (2009). Molecular and biochemical characterization of the tetralin degradation pathway in *Rhodococcus* sp. strain TFB. Microb Biotechnol.

[b47] Tomás-Gallardo L, Santero E, Floriano B (2012). Involvement of a putative cyclic amp receptor protein (CRP)-like binding sequence and a CRP-like protein in glucose-mediated catabolite repression of thn genes in *Rhodococcus* sp. strain TFB. Appl Environ Microbiol.

[b48] Yen KM, Serdar CM (1988). Genetics of naphthalene catabolism in Pseudomonads. Crit Rev Microbiol.

[b49] Yuste L, Hervás AB, Canosa I, Tobes R, Jiménez JI, Nogales J (2006). Growth phase-dependent expression of the *Pseudomonas putida* KT2440 transcriptional machinery analysed with a genome-wide DNA microarray. Environ Microbiol.

[b50] Zhang JJ, Liu H, Xiao Y, Zhang XE, Zhou NY (2009). Identification and characterization of catabolic para-nitrophenol 4-monooxygenase and para-benzoquinone reductase from *Pseudomonas* sp. strain WBC-3. J Bacteriol.

[b51] Zhou NY, Fuenmayor SL, Williams PA (2001). *nag* genes of *Ralstonia* (formerly *Pseudomonas*) sp. strain U2 encoding enzymes for gentisate catabolism. J Bacteriol.

